# Yoda1 pretreated BMSC derived exosomes accelerate osteogenesis by activating phospho-ErK signaling via Yoda1-mediated signal transmission

**DOI:** 10.1186/s12951-024-02669-0

**Published:** 2024-07-10

**Authors:** Xi He, Yanling Liu, Zhongyu Dai, Yu Chen, Wenbin Liu, Honglian Dai, Yihe Hu

**Affiliations:** 1 Department of Orthopedics, The First Affiliated Hospital, Zhejiang University School of medicine, Hangzhou, 310002 China; 2 School of Basic Medical Sciences and Forensic Medicine, Hangzhou Medical College, Hangzhou, Zhejiang China; 3 Department of Orthopedics, The Third Xiangya Hospital, Central South University, Changsha, 410078 China; 4 Hunan Engineering Research Center of Biomedical Metal and Ceramic Implants, Changsha, China; 5 Biomedical Materials and Engineering Research Center of Hubei Province, Wuhan University of Technology, Wuhan, 430070 China

**Keywords:** Osteogenesis, Yoda1, Bone defect repair, Hydrogels, Exosome, Extracellular vesicles

## Abstract

Segmental bone defects, arising from factors such as trauma, tumor resection, and congenital malformations, present significant clinical challenges that often necessitate complex reconstruction strategies. Hydrogels loaded with multiple osteogenesis-promoting components have emerged as promising tools for bone defect repair. While the osteogenic potential of the Piezo1 agonist Yoda1 has been demonstrated previously, its hydrophobic nature poses challenges for effective loading onto hydrogel matrices.In this study, we address this challenge by employing Yoda1-pretreated bone marrow-derived mesenchymal stem cell (BMSCs) exosomes (Exo-Yoda1) alongside exosomes derived from BMSCs (Exo-MSC). Comparatively, Exo-Yoda1-treated BMSCs exhibited enhanced osteogenic capabilities compared to both control groups and Exo-MSC-treated counterparts. Notably, Exo-Yoda1-treated cells demonstrated similar functionality to Yoda1 itself. Transcriptome analysis revealed activation of osteogenesis-associated signaling pathways, indicating the potential transduction of Yoda1-mediated signals such as ErK, a finding validated in this study. Furthermore, we successfully integrated Exo-Yoda1 into gelatin methacryloyl (GelMA)/methacrylated sodium alginate (SAMA)/β-tricalcium phosphate (β-TCP) hydrogels. These Exo-Yoda1-loaded hydrogels demonstrated augmented osteogenesis in subcutaneous ectopic osteogenesis nude mice models and in rat skull bone defect model. In conclusion, our study introduces Exo-Yoda1-loaded GELMA/SAMA/β-TCP hydrogels as a promising approach to promoting osteogenesis. This innovative strategy holds significant promise for future widespread clinical applications in the realm of bone defect reconstruction.

**Graphical Abstract:**

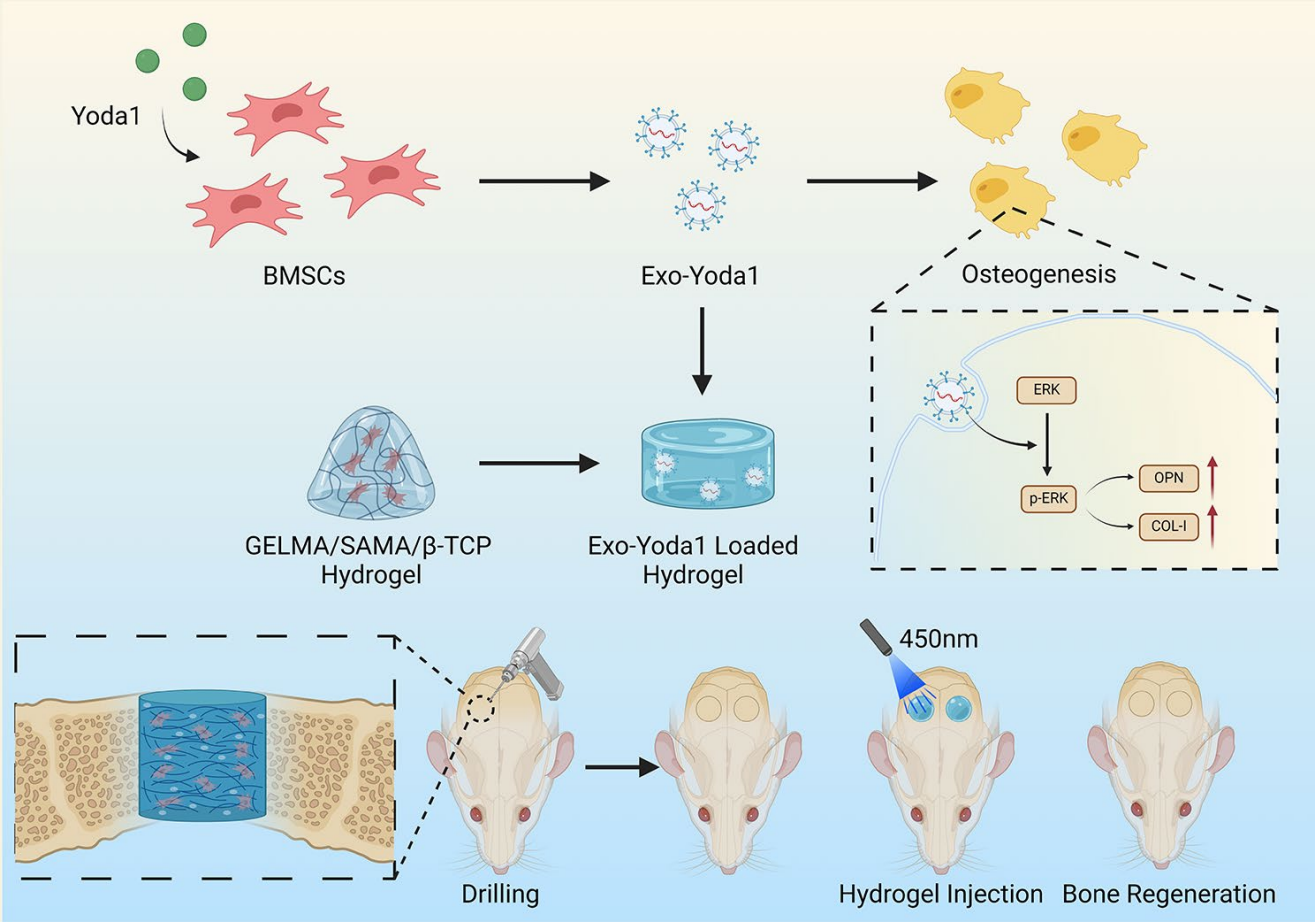

**Supplementary Information:**

The online version contains supplementary material available at 10.1186/s12951-024-02669-0.

## Highlights

• Exosomes derived from Yoda1 pretreated BMSCs promoted osteogenesis by transducing biological signals activated by Yoda1.
• GELMA/SAMA/β-TCP hydrogels loading with Exo-Yoda1 was highly bio-compatibility and suitable for massive commercial application.

**Supplementary Information:**

The online version contains supplementary material available at 10.1186/s12951-024-02669-0.

## Introduction

Segmental bone defects, arising from factors such as trauma, tumor resection, and congenital malformations, pose significant clinical challenges that often necessitate reconstruction. This is typically achieved through the utilization of autologous or allogeneic bone grafts, as well as artificial synthetic bone materials. The growing resilience exhibited by artificial synthetic bone scaffold materials underscores their promising potential for widespread clinical application [1]. Among these materials, hydrogels have emerged as particularly notable due to their myriad advantages. Hydrogels possess an extracellular matrix (ECM)-mimicking cross-linked three-dimensional structure, fostering an environment conducive to cell adhesion, proliferation, migration, and differentiation. When integrated with suitable support frameworks, hydrogels exhibit the potential to significantly augment the mending of crucial bone defects. The polymer network of hydrogels has the capacity to accommodate various bioactive agents, encompassing exosomes, siRNA, and similar entities, which play a pivotal role in stimulating osteogenesis and facilitating the bone repair process [2].

Extracellular vesicles (EVs) refer to a collective term for various lipid bilayer membrane structures released by cells. Based on differences in EV generation, size, or function, they can be categorized into different subgroups such as exosomes, microvesicles, and apoptotic bodies. Exosomes are small, particle-like vesicles with a diameter of 30–150 nm formed by budding inward [3]. Previous research has unequivocally demonstrated the efficacy of exosomes in facilitating the process of bone repair.Exosomes derived from sources such as dexamethasone, osteogenic induction conditioned medium [4], and Curcumin-preconditioned Mesenchymal Stem Cells (MSCs) exhibit a remarkable capacity to stimulate osteogenesis [5]. These exosomes play a pivotal role in orchestrating bone repair mechanisms through signal transduction. Moreover, exosomes obtained from MSCs that have undergone hypoxia pretreatment offer a noteworthy avenue for bolstering osteogenesis and augmenting the bone repair process. This effect is corroborated by findings indicating an augmented angiogenic response in hypoxia-treated cells [6, 7]. Furthermore, exosomes have demonstrated the ability to transduce mechanical strain forces, thereby fostering osteogenic differentiation in hPDLSCs via the BMP2/Runx2 signaling pathway [8]. Other bioengineering extracellular vesicles also functioned well in vivo and vitro [9–13].

Mechanical loading exerted upon the skeletal framework emerges as a pivotal determinant of bone development, growth, and upkeep. The stimulation of the Piezo1 channel’s transport mechanism elicits a transformation into biological signals, thereby assuming a critical role in both osteogenesis and osteogenic differentiation. Notably, perturbing the expression of Piezo1 in murine models has been correlated with the onset of osteoporosis and the occurrence of spontaneous fractures [14, 15]. Conversely, mechanical stimuli encompassing techniques like LIPUS, hydrostatic pressure, and shear stress have been shown to potentiate osteogenic differentiation through the activation of Piezo1 [16–18]. Intriguingly, even exosomes obtained from MSCs subjected to mechanical stimulation have exhibited a propensity to promote osteogenesis [8]. Moreover, exosomes originating from MSCs treated with cyclic mechanical stretching have demonstrated the capacity to impede RANKL-induced osteoclastogenesis [19]. The osteogenic potential of the Piezo1 agonist Yoda1 has also been validated [16, 20]. Nonetheless, the considerable hydrophobicity of Yoda1 has posed challenges in achieving efficient concentrations when loaded onto hydrogels. As of now, only limited investigations have addressed the prospect of Yoda1-pretreated exosomes engendering osteogenesis by translating mechanical cues into bioactive signals within recipient cells.

Thus, this study endeavors to introduce a paradigm wherein GELMA/SAMA/β-TCP hydrogels are loaded with exosomes derived from Yoda1-pretreated BMSCs (Exo-Yoda1). GELMA/SAMA/β-TCP hydrogels were constituted with 𝛽-tricalcium phosphate (𝛽-TCP, calcium supplier) as well as different proportions of methacrylated sodium alginate (SAMA) and gelatin methacryloyl (GelMA).

Our investigations have unequivocally substantiated the osteogenic potential of Exo-Yoda1, rivaling the efficacy of Yoda1 itself in cellular models. Remarkably, hydrogels imbued with Exo-Yoda1 have demonstrated superior capabilities in rectifying bone defects. To conclude, this study pioneers a novel approach to bone defect remediation, overcoming the limitations inherent to the hydrophobic Yoda1 while proficiently transducing mechanical signals into consequential bioactive responses.

## Results

### Characteristics of exosomes

**Fig. 1:**
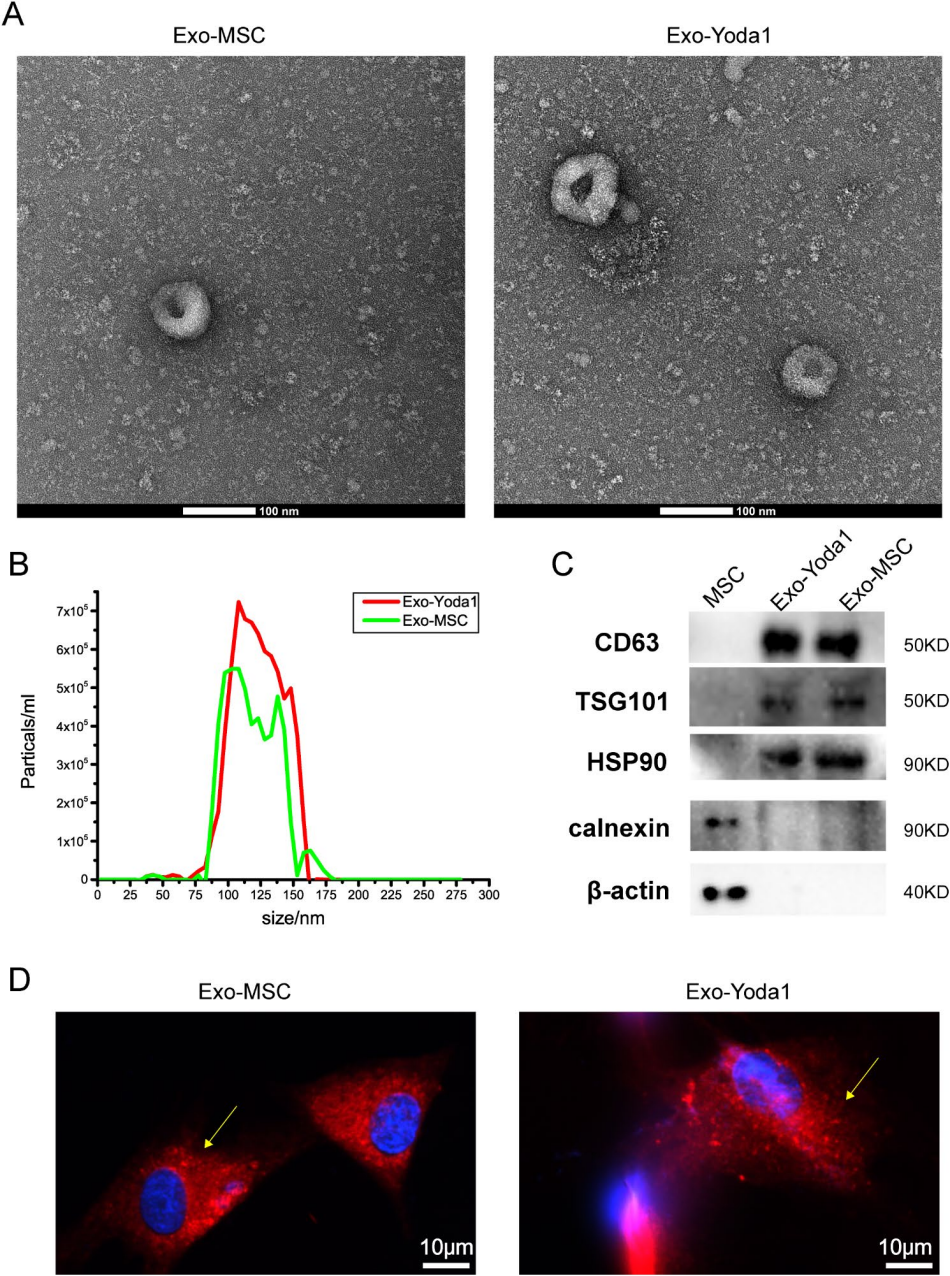
Identification of Exosomes (**A**) morphology of Exo-MSC and Exo-Yoda1 detected by TEM, the scale was 100 nm; (**B**) particle size distribution of Exo-MSC and Exo-Yoda1 by NTA; (**C**) expression of CD63, TSG101, HSP90, β-actin and marker calnexin by WB; (**D**) the fluorescence of Dil, the yellow arrow indicated positive Dil stain in BMSCs, the scale was 10 μm

Utilizing transmission electron microscopy (TEM), the circular vesicles constituting Exo-MSC and Exo-Yoda1, pivotal entities in this inquiry, were discerned. These vesicles were enveloped by a dual-layer membrane and demonstrated an approximate diameter of 100 nm (Fig. 1A). Through nanoparticle tracking analysis (NTA), a comprehensive appraisal of particle size distribution unveiled a consistent range spanning 80 to 155 nm for both Exosomes (Fig. 1B). Furthermore, the analysis encompassed the identification of exosomal markers, notably CD63, HSP90, and TSG101, solidifying the character of the exosomes. Conversely, no expression of β-actin or the endoplasmic reticulum (ER) marker calnexin was detected within the exosomes isolated for the current study, as delineated (Fig. 1C). In a subsequent engagement involving the incubation of Exo-MSC^dil^ and Exo-Yoda1^dil^ with BMSCs for a duration of 4 h, positive staining for Dil was showed in the BMSCs, thus affirming the successful endocytic uptake of the exosomes (Fig. 1D).

### Exo-Yoda1 promote osteogenesis in BMSCs

**Fig. 2:**
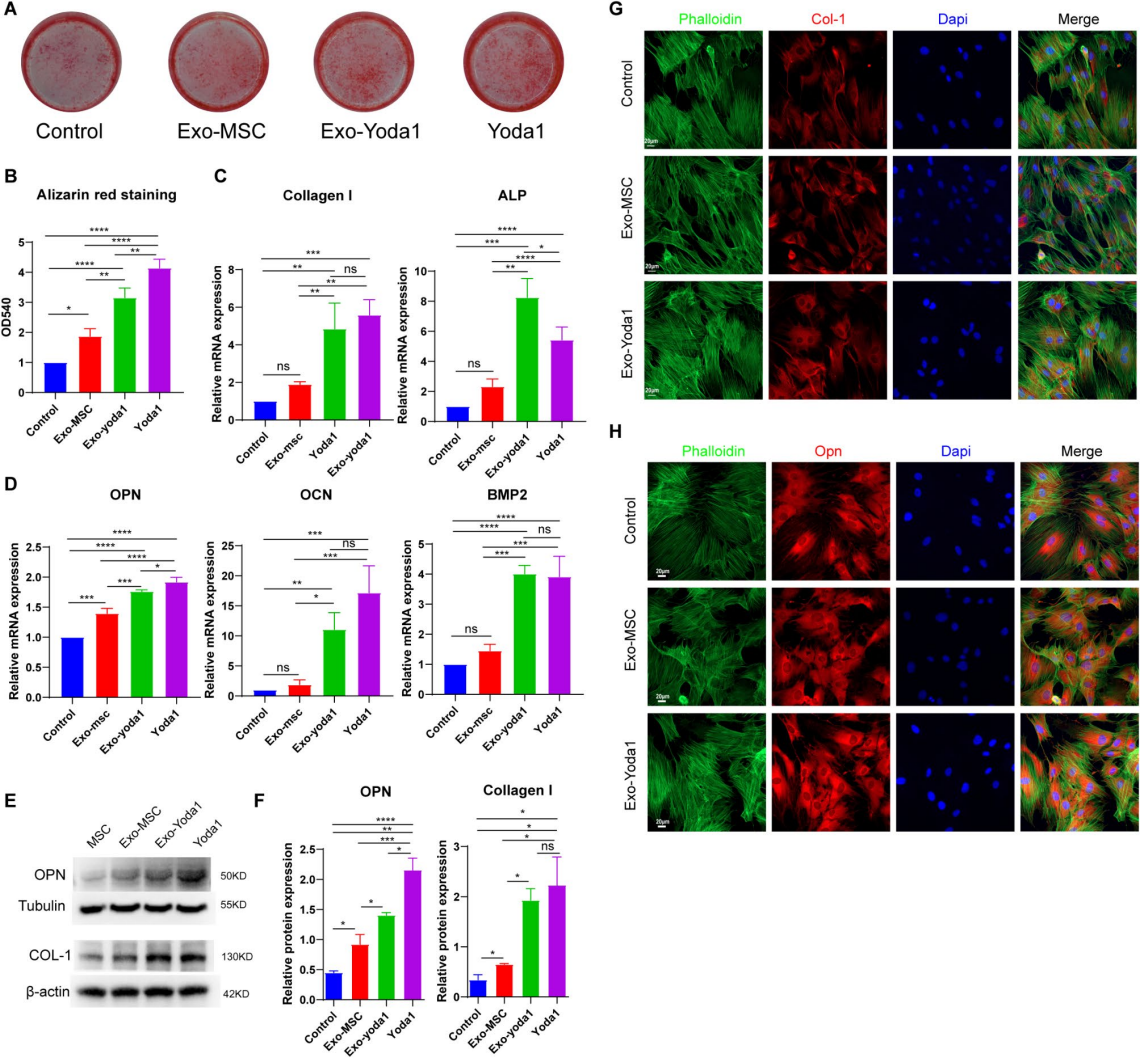
>Exo-Yoda1 promote osteogenesis in BMSCs (**A**) Alizarin red (lower) staining of control and treated groups; (**B**) Quantification of ARS; The expression of Collagen I, ALP (**C**), OPN, OCN and BMP2 (**D**) detected by qPCR; (**E**) the expression of OPN and Collagen I detected by WB; (**F**) Density analysis of OPN and Collagen I; (**G**) and (**H**), the expression of COL-1(in red), OPN (in red) and Phalloidin (in green) in BMSCs, the scale is 20 μm; * *p* < 0.5, **p < 0.01, *** *p* < 0.001, **** *p* < 0.0001

Through the direct incorporation of Exosomes and Yoda1 into osteogenic-inducing media, co-cultured with BMSCs, the emergence of alkaline phosphatase (ALP), an early hallmark of osteogenesis, was detected in day 5. Upon comparative analysis with control groups, it became evident that Exo-MSC, Exo-Yoda1, and Yoda1 cohorts manifested heightened staining intensity, indicative of elevated osteogenic activity (Supplementary Figure S1A). Particularly noteworthy were the Exo-Yoda1 and Yoda1 groups, which exhibited the most pronounced staining depth. Moving forward to day 14, alizarin red staining was performed, revealing a conspicuous augmentation in calcium deposition within the Exo-MSC, Exo-Yoda1, and Yoda1 groups (Fig. 2A and B). While both the Exo-Yoda1 and Yoda1 groups displayed heightened deposition in comparison to the Exo-MSC group, and Yoda1 seemed had more deposition than Exo-Yoda1 group.

Osteogenesis-associated gene expression was assessed through qPCR and WB after the initiation of differentiation induction (Fig. 2C-F). In concordance with the outcomes garnered from ALP and alizarin red staining, discernible elevations in ALP expression were detected within the Exo-MSC, Exo-Yoda1, and Yoda1 groups, in comparison to the Control group, as demonstrated (Fig. 2C and D). Particularly noteworthy is the observation that both the Exo-Yoda1 and Yoda1 groups exhibited heightened ALP expression surpassing that of the Exo-MSC group. However, discerning differences between the Exo-Yoda1 and Yoda1 groups were only found in the expression of OPN (Fig. 2D-F). While the Exo-Yoda1 group demonstrated elevated ALP expression in comparison to the Yoda1 group, it simultaneously exhibited a comparatively diminished osteocalcin (OCN) expression. The findings obtained from Western blot analysis (WB) aligned harmoniously with those deduced from qPCR assessments (Fig. 2E and F).

Morphological assessments (using phalloidin) and the evaluation of Collagen I (Col-1) expression were conducted within BMSCs after differentiation 7 days (Fig. 2G), along with the scrutiny of Osteopontin (OPN) expression (Fig. 2H). The trends in fluorescence intensity observed for Col-1 and OPN mirrored the findings derived from Western blot analysis. It is noteworthy that the Phalloidin staining indicated a heightened structural integrity in the Exo-Yoda1 group, surpassing that of the remaining two groups.

Given the significance of BMSCs migration in facilitating osteogenesis during fracture healing, we undertook an investigation to ascertain whether Exo-Yoda1 or Yoda1 could potentiate migratory responses (supplementary Figure S1B). Our findings reveal that, in comparison to control groups, both Exo-MSC (*p* < 0.05) and Exo-Yoda1 (*p* < 0.0001), as well as Yoda1 (*p* < 0.0001), exhibited a statistically significant enhancement in migration. Upon a comparative analysis of the Exo-MSC, Exo-Yoda1, and Yoda1 groups, both Exo-Yoda1 (*p* < 0.05) and Yoda1 (*p* < 0.05) demonstrated heightened migratory capacity when juxtaposed with the Exo-MSC group (supplementary Figure S1C). This observation implies that Exo-Yoda1 could potentially wield a migratory influence akin to that of Yoda1. Notably, no substantial disparities were detected between the Exo-Yoda1 and Yoda1 groups, thereby hinting at the likelihood of a comparable migratory effect for Exo-Yoda1 as exhibited by Yoda1.

Therefore, our findings suggested that Exo-Yoda1 can assume a role akin to Yoda1 in fostering osteogenic differentiation, and it offered the added advantage of heightened biocompatibility.

### Exo-Yoda1 promoted osteogenesis through ErK signaling pathways

**Fig. 3:**
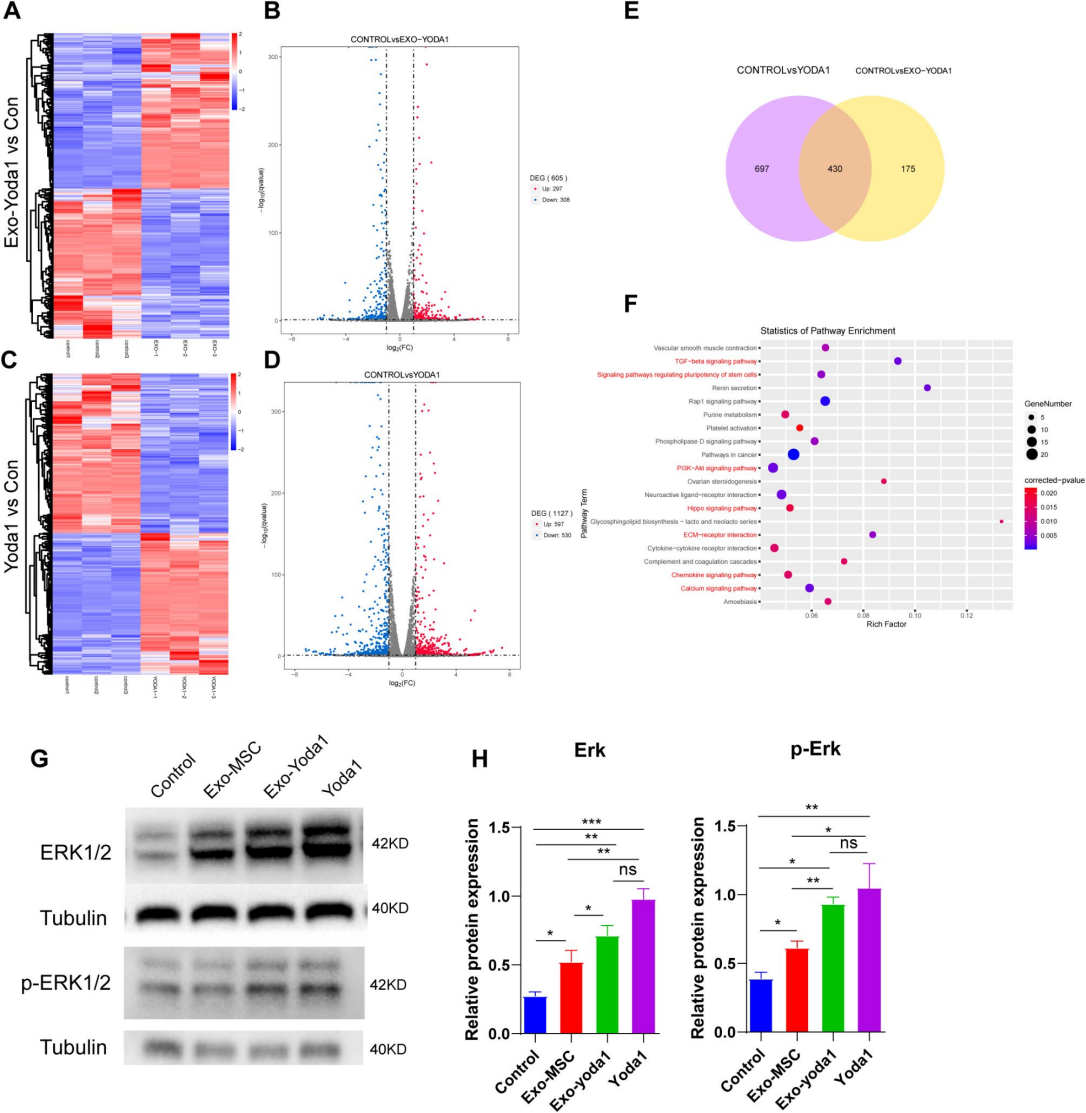
Gene expression changes in Exo-Yoda1 pretreated BMSCs and the verification of RNA-Seq results. (**A**) Heatmap of top 200 genes differentially expressed between Exo-Yoda1 VS Control; (**B**) Volcano map of differentially expressed genes in Exo-Yoda1; (**C**) Heatmap of top 200 genes differentially expressed between Yoda1 VS Control; (**D**) Volcano map of differentially expressed genes in Yoda1; (**E**) Venn diagram depicting the overlap differentially expressed genes between Exo-Yoda1 and Yoda1; (**F**) Cluster of consensus overlapped differentially expressed genes; (**G**) by WB, we observed increasing ErK1/2 and phosphate- ErK1/2 in Exo-Yoda1 and Yoda1 group; (**H**) Density analysis of ErK and p-ErK; * *p* < 0.5, **p < 0.01, *** *p* < 0.001, **** *p* < 0.0001

To further explore how Exo-Yoda1 promote osteogenesis, transcriptome analysis was applied. RNA was isolated as described in Method and transcriptome analysis was conducted. Supervised hierarchical clustering based on the top 200 differential expressed genes showed distinct clustering of Exo-Yoda1 group VS Control (Fig. 3A), as well as Yoda1 vs. Control (Fig. 3C). We identified 605 genes showing significant expression changes in Exo-Yoda1, including 297 high expressed and 308 down expressed (Fig. 3B). We also identified 1127 differentially expressed genes in Yoda1, including 597 high expressed and 530 down expressed (Fig. 3D).

KEGG pathway enrichment analysis was conducted on transcriptome data. In both Exo-Yoda1 and Yoda1, pathways associated with osteogenesis differentia including PI3K-Akt signaling pathway, TGF-beta signaling pathway, signaling pathways regulating pluripotency of stem cells, ECM-receptor interaction, neuroactive ligand-receptor interaction were up regulated, which indicated Exo-Yoda1 could enhance osteogenesis and the ability to repair bone defects (Supplementary Figure S2A and S2B). We also conducted GO (gene ontology analysis) analysis based on Exo-Yoda1 and Yoda1 data. GO analysis also identified similar signaling pathways in both Exo-Yoda1 and Yoda1. Which was associated with osteogenesis (Supplementary Figure S2C and S2D).

We observed a highly significant overlap Yoda1 and Exo-Yoda1 (Fig. 3E). There were 430 overlapped differentially expressed genes between Yoda1 and Exo-Yoda1, which cluster into signaling pathways including TGF-beta signaling pathway, PI3K-Akt signaling pathway, signaling pathways regulating pluripotency of stem cells, ECM-receptor interaction, Chemokine signaling pathway, Calcium signaling pathway (Fig. 3F). The signal pathways related to the two sets of shared differentially expressed genes involve the classical osteogenic pathway, which is closely related to osteogenic differentiation. Moreover, the proportion of shared differentially expressed genes in the Exo-Yoda1 and Yoda1 group was high.

To verified this assumption, we searched for pathways and key regulated molecular which were consensus in Exo-Yoda1 and Yoda1, as well as in the downstream of Piezo1. Through GO analysis, both Exo-Yoda1 and Yoda1 clustered in regulation of ErK1 and ErK2 cascade (Supplementary Table S2), and ERK1/2 was proved to be regulated by Piezo1 [21]. By incubated with Exo-MSC, Exo-Yoda1 and Yoda1 for 24 h, we observed significant increasing in ErK1/2 and phosphate- ErK1/2 compared with both Control and Exo-MSC, which inferred that Exo-Yoda1 could transmit the signals activated by Yoda1 (Fig. 3G and H).

### The characteristics of exosomes loaded GELMA/SAMA/β-TCP

**Fig. 4:**
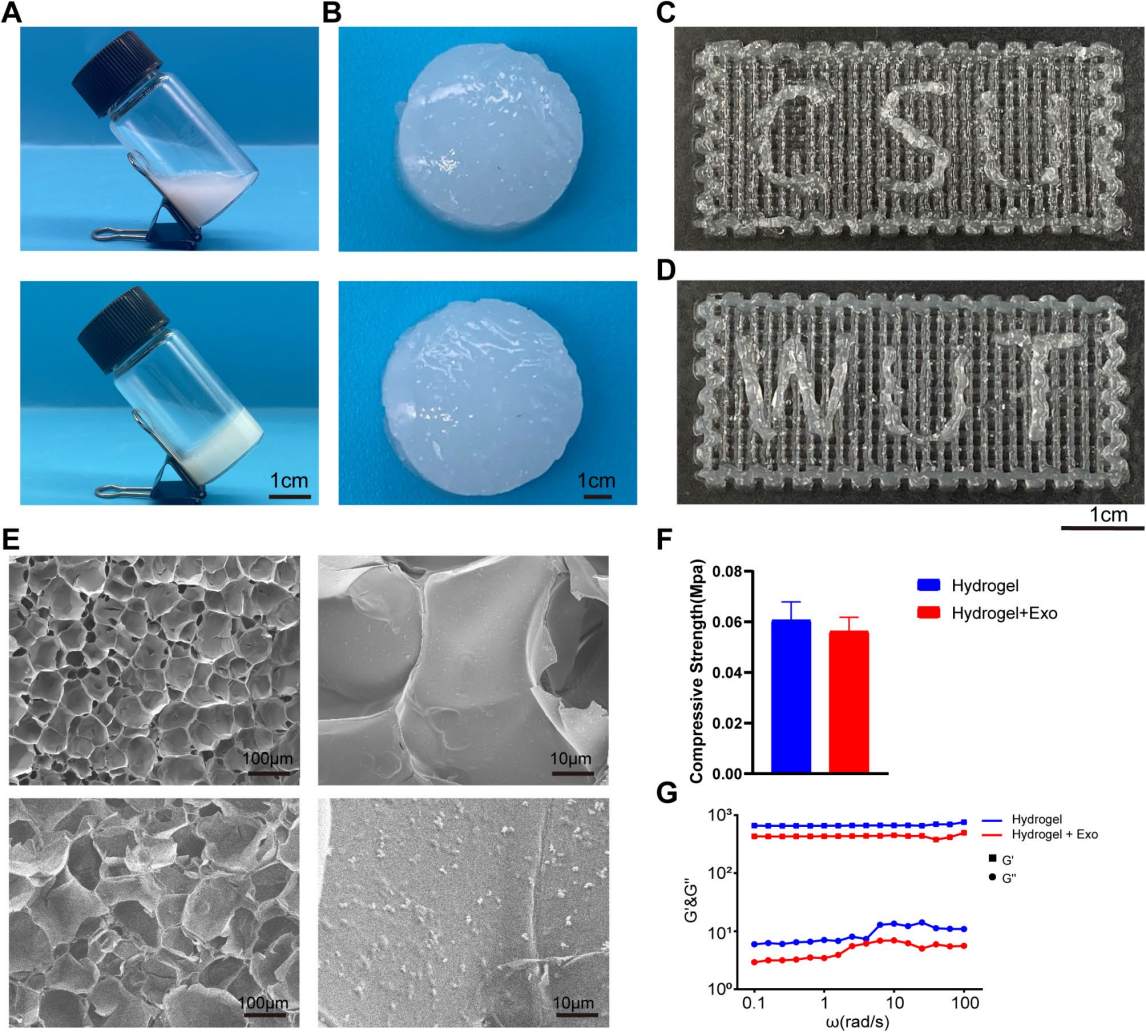
Characteristics of GELMA/SAMA/β-TCP loaded Exosomes (**A**) solid (lower) other than liquid (upper) gels sticked to the bottom of the tilt; (**B**) gels loaded with exosomes (upper) and without exosomes (lower) had the same appearance; (**C**) and (**D**), gels loaded with exosomes (**C**) and without exosomes (**D**) can be easily modified to multiple shapes; (**E**) by SEM, the structure of both hydrogels seemed identical expected small dots of exosomes observed in Hydrogels with exosomes; (**F**) compressive strength of both hydrogels was the same; (**G**) the gel time of both hydrogels was the same

By introducing modifications to the carbon-carbon double bond within the photosensitive group of the matrix material, the GELMA/SAMA/β-TCP hydrogels exhibited the ability to establish interconnected networks under blue light irradiation. This illumination effectively stimulated the photoinitiator LAP, inducing the generation of active free radicals that initiated polymerization through carbon-carbon double bonds. A tilt test was conducted, revealing the hydrogels adhering to the vial’s base as they transitioned from a liquid state to a solid one under light-induced crosslinking (Fig. 4A). Post-molding, hydrogels presented an indistinguishable appearance whether loaded with or without exosomes (Fig. 4B). The utilization of a bluelight-cured 3D printer facilitated facile manipulation of hydrogels, regardless of exosome incorporation, into diverse geometries (Fig. 4C and D). Employing SEM, the examination of hydrogel microstructure exhibited consistent porous arrangements characterized by analogous pore dimensions, regardless of exosome presence. Notably, upon magnification, discernible dot structures surfaced within exosome-laden gels (Fig. 4E).

Subsequent evaluation encompassed the compressive strength analysis of the two hydrogel formulations. Despite a minor decline in compressive strength observed in exosome-loaded hydrogels, this reduction remained statistically insignificant (Fig. 4F). To gauge the critical parameter of gelation time, pivotal in the context of tissue engineering scaffolds, a rotary rheometer was employed. The outcomes conclusively revealed that no appreciable divergence existed in the gelation kinetics and temporal progression between the two hydrogel variations (Fig. 4G).

### Sustained release of hydrogel loaded exosomes in vivo

**Fig. 5:**
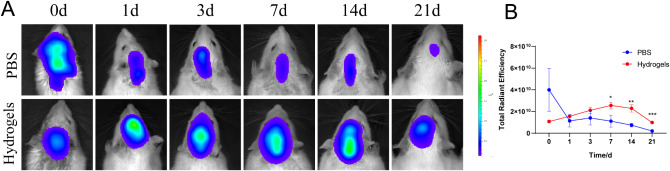
Sustained releasing of hydrogel loaded exosomes in vivo. (**A**) DiR fluorescence images of PBS and Hydrogel groups from day 0 to day 21 after surgery; (**B**) releasing curve of PBS and Hydrogel groups (down); * *p* < 0.5, **p < 0.01, *** *p* < 0.001, **** *p* < 0.0001

Hydrogels imbued with DiR-labeled exosomes (referred to as exosomes^DiR^) were introduced subcutaneously into Rat, while exosomes^DiR^ diluted with PBS buffer served as the positive control. In the initial 24 h post-implantation (day 0–1), the PBS control group exhibited an initial surge in exosomes^DiR^ concentration. By the subsequent day after implantation, the hydrogel-treated groups demonstrated notably heightened fluorescence intensity compared to the PBS control, a trend that persisted for the ensuing 21 days. Conversely, the fluorescence signal within the PBS control group progressively attenuated, becoming undetectable by day 21 (Fig. 5A). Notably, the sustained release profile of the hydrogel group exhibited a consistent plateau, sustaining a high exosomal concentration over an extended duration. Further analysis of the sustained release kinetics revealed a significantly elevated fluorescence signal within the hydrogel group compared to the PBS group from day 7 through day 21 (Fig. 5B). These findings collectively underscore the hydrogel’s capacity to facilitate the sustained release of exosomes, thus maintaining a gradual and protracted exosomal release profile in vivo, thereby extending the duration and augmenting the therapeutic effect.

### Exo-Yoda1 loaded hydrogels promote osteogenesis

**Fig. 6:**
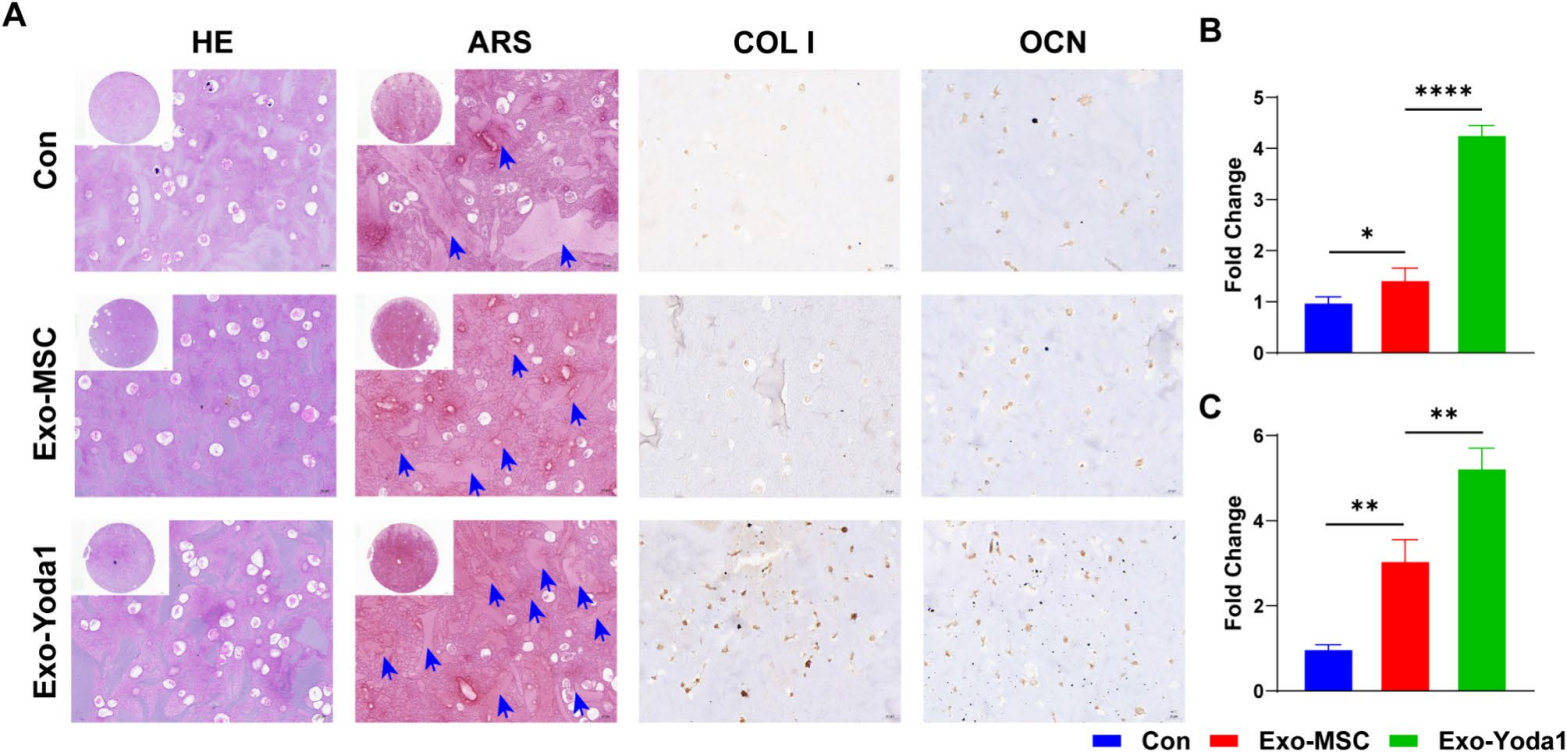
Exo-Yoda1 promote osteogenesis in vitro. (**A**) By H&E staining, it was shown that Exo-Yoda1 group had the most alive cells; By ARS staining, we observed the most collagen deposition in Exo-Yoda1 group, blue arrows point to calcium nodules for a darker red color; we also observed the highest expression of Collagen I and OCN in Exo-Yoda1 group; (**B**) the quantification analysis of COL1; (**C**) the quantification analysis of OCN, * *p* < 0.5, **p < 0.01, *** *p* < 0.001, **** *p* < 0.0001

Hydrogels mixed with BMSCs and Exosomes were cultured in osteogenic media for 21 days. We observed osteogenesis and collagen deposition in Exo-Yoda1 hydrogels as well as higher expression of Collagen I and OCN (Fig. 6A-C).

**Fig. 7:**
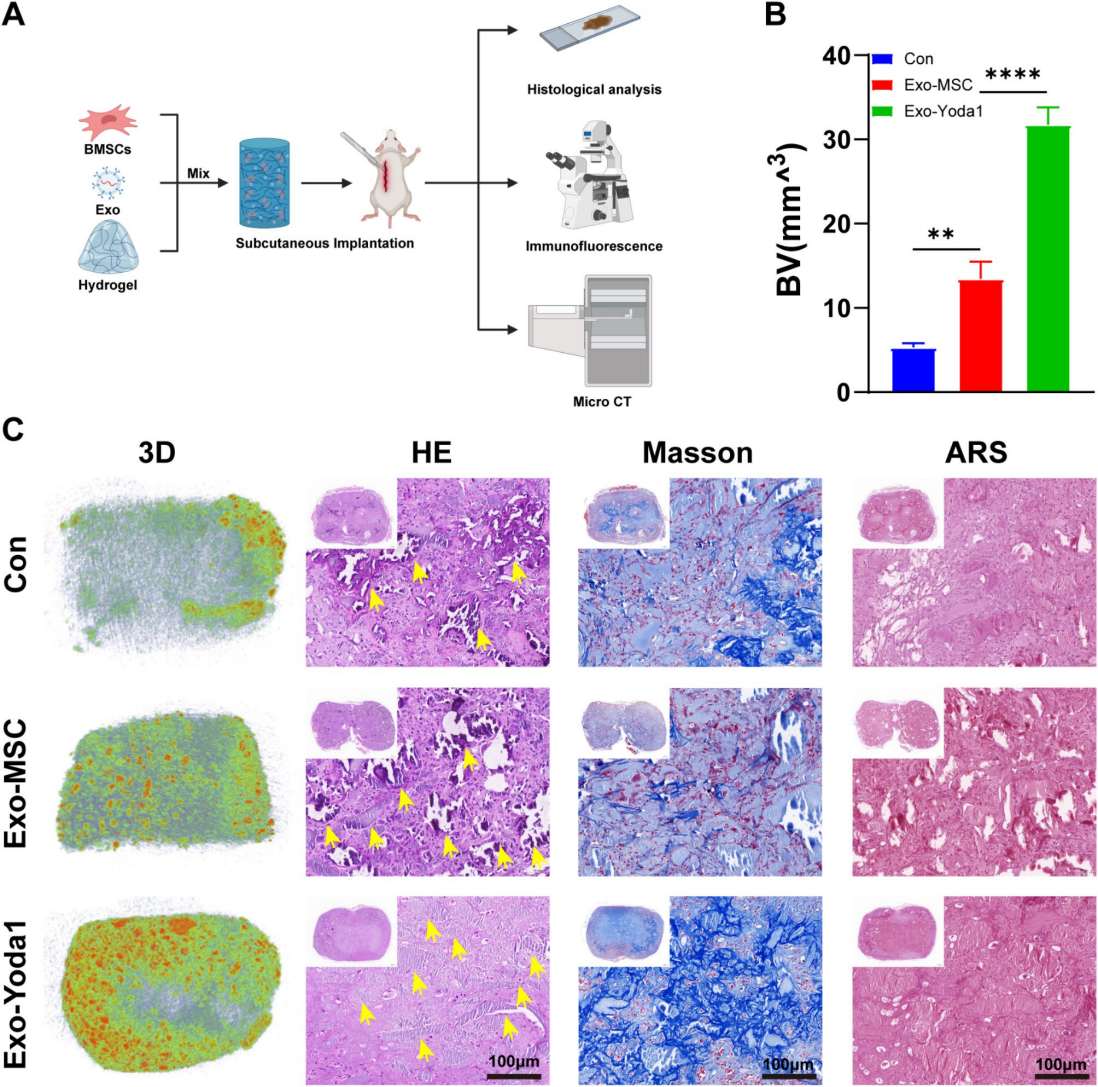
Exo-Yoda1 promote osteogenesis in subcutaneous ectopic osteogenesis nude mice models (**A**) graphical description of subcutaneous ectopic osteogenesis nude mice models; (**B**)Statistical analysis of BV among groups; (**C**) H&E, Masson, and Alizarin red staining in subcutaneous ectopic osteogenesis, the scale was 100 μm, the yellow arrows indicated crystal-like calcium nodules, * *p* < 0.5, **p < 0.01, *** *p* < 0.001, **** *p* < 0.0001

Hydrogels mixed with BMSCs and Exosomes were implanted under the skin of nude mice (Fig. 7A). We observed osteogenesis (Fig. 7C and Supplementary Figure S3), collagen deposition (Fig. 7C) and vascular ingrowth (Supplementary Figure S3) inside the hydrogels, while no obvious inflammatory cell infiltration was detected by H&E staining (Fig. 7C), which indicated high bio-compatibility of hydrogels. By Masson and Alizarin red staining (Fig. 7C), it was indicated collagen and calcium deposition accumulated the most in that Exo-Yoda1 group. Although Exo-MSC also promoted collagen and calcium deposition, however, it was still inferior to Exo-Yoda1 group (Fig. 7B).

**Fig. 8:**
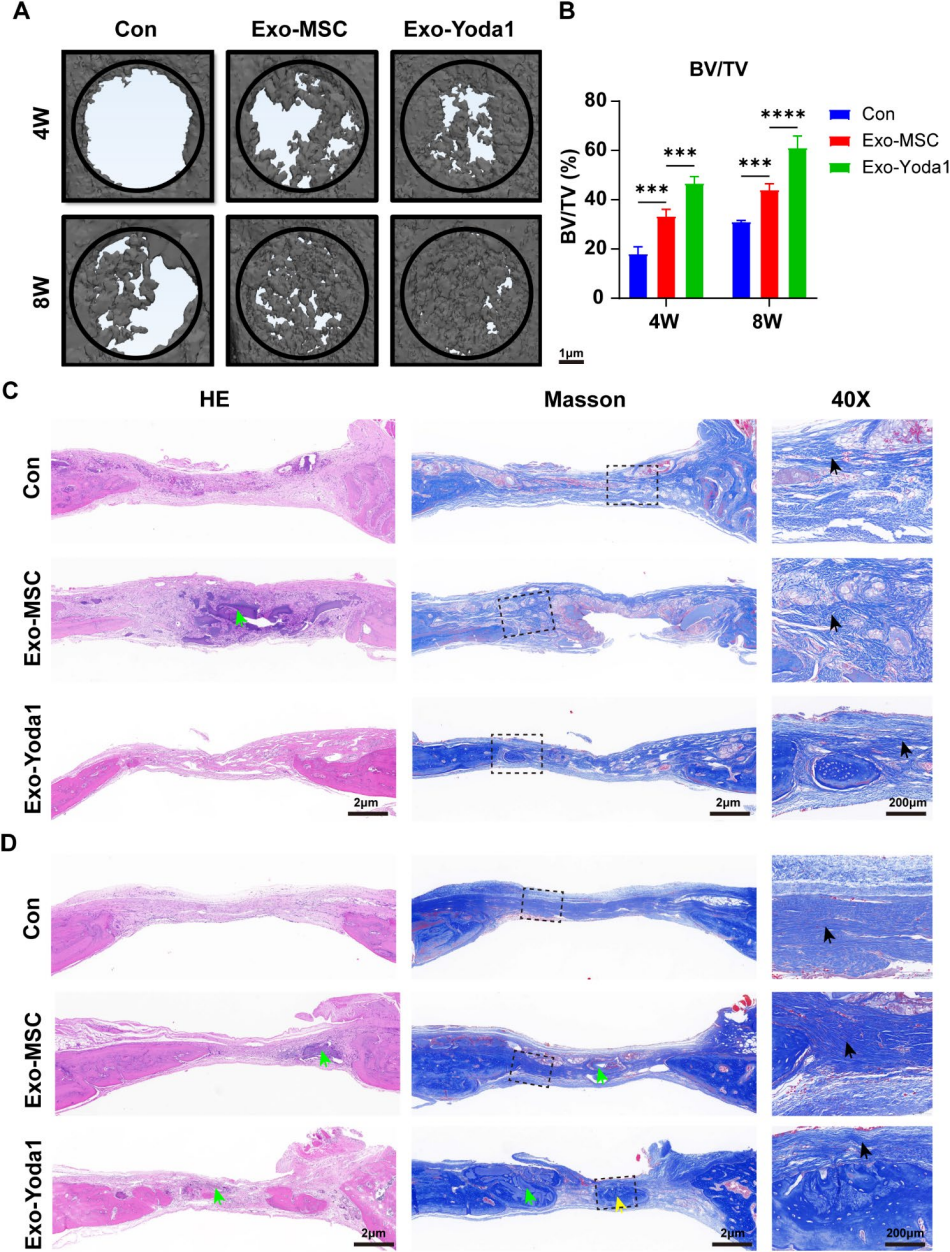
Exo-Yoda1 hydrogels promoted osteogenesis in bone defect of rat skull model (**A**) By micro-CT, Exo-Yoda1 group showed the best repairment; (**B**) analysis of BV/TV; (**C**) and (**D**), H&E and Masson staining of samples collected 4 weeks (**C**) and 8 weeks (**D**) after surgery; the green arrows indicated un-degradation hydrogels, the yellow arrows indicated new bones, the black arrows indicated collagens, the squares indicated magnification sites; * *p* < 0.5, **p < 0.01, *** *p* < 0.001, **** *p* < 0.0001

We procured specimens at intervals of 4 and 8 weeks subsequent to the surgical intervention, utilizing a rat skull model featuring a bone defect. Employing micro-CT scanning and a meticulous analysis of the bone trabecular fraction (BV/TV), it became evident that the cohorts treated with exosomes exhibited heightened reparative tendencies at both the 4-week and 8-week junctures, in contrast to the control group. Notably, when the exosomes were categorized into Exo-MSC and Exo-Yoda1 groups, the latter manifested a more pronounced proclivity for repairment, a finding that correlated concomitantly with the osteogenesis-related outcomes observed earlier within the confines of this investigation (Fig. 8A and B). Employing Masson and H&E staining techniques, we ascertained the continued presence of hydrogels even at the 8-week mark subsequent to the surgical intervention. Remarkably, the Exo-Yoda1 group evinced the most conspicuous collagen deposition, along with the most discernible bone tissue architecture among all the groups (Fig. 8C and D, Figure S3). In 4-week samples, we found disordered collagen fibers, however, in 8-week samples, we identified well-organized collagen fiber bundles in defecting areas.

## Discussion

In this study, it was observed that Exo-Yoda1 exhibited osteogenic attributes akin to those of Yoda1. Notably, the hydrogel incorporating Exo-Yoda1 demonstrated a heightened propensity for osteogenesis when juxtaposed with the Exo-MSC counterpart. This augmentation in osteogenic potential was substantiated through comprehensive assessments involving both cellular and animal models, thereby affirming its efficacy in the realm of bone defect remediation. It can be inferred that Exo-Yoda1 can transmit some of the effects of Yoda1, which also means that Exo-Yoda1 can transmit some of the mechanical signal channels of Piezo1.

Yoda1, a hydrophobic compound, functions as an agonist of Piezo1. In previous investigations, hydrophobic compounds were incorporated into guided bone regeneration membranes, commonly utilizing collagen as the substrate. Nonetheless, the utilization of such membranes has been impeded by rapid degradation and cost constraints [22]. A recent study sought to address this limitation by developing a bilayer membrane comprising PLGA and collagen loaded with Yoda1, yielding favorable osteogenic effects [23]. Despite this advancement, the widespread adoption of bilayer membranes has been hindered by the intricacies associated with their fabrication process. Considering the limitation of transmitting Yoda1 directly, we tried to using Exo-Yoda1 to transmitting Yoda1 signaling indirectly. Exosomes present a host of advantageous attributes, including compatibility with living tissues, negligible toxicity, prolonged systemic circulation, intercellular cargo transfer capability, non-immunogenic properties, and specialized cell targeting capabilities, rendering them exceptionally promising therapeutic vectors [10, 11, 24]. In the present investigation, Exo-Yoda1 was derived from Yoda1-pretreated MSCs, ensuring heightened biocompatibility, with the extraction procedures being based on established commercial protocols conducive to large-scale applications. The hydrogel platforms devised in this study exhibited commendable potential as vehicles for the gradual release of exosomes, simultaneously furnishing a scaffold conducive to bone regeneration. Notably, the GELMA/SAMA/β-TCP hydrogels employed herein are amenable to 3D printing techniques, thus poised to facilitate the creation of varied morphologies tailored to diverse bone defect profiles in subsequent endeavors.

Previous studies showed Piezo1 promoted osteogenesis through activating multiple signaling pathways including Hippo-YAP/TAZ [15, 25, 26], MAPK (JNK, p38, ErK), TGF-β, NFAT-YAP1-ß-catenin, PI3K-AKT [17, 21, 27]. The cascades downstream of MAPK and PI3K-AKT culminate in the activation of ErK1/2 [28], prominent proteins pivotal to osteogenic and angiogenic processes. In the present investigation, we discerned disparities within the PI3K-AKT pathway via comprehensive transcriptome analysis, aligning with findings from prior studies wherein Yoda1 was administered directly to cells, as opposed to its exosomal counterpart, Exo-Yoda1. Heightened levels of ErK1/2 were evident in both the Exo-Yoda1 and Yoda1 experimental groups. Consequently, we posit that Exo-Yoda1 exhibits analogous functionality to Yoda1, substantiating their equivalence in effect.

## Conclusion

Our study introduces Exo-Yoda1-loaded GELMA/SAMA/β-TCP hydrogels as a promising approach to promoting osteogenesis. This innovative strategy holds significant promise for future widespread clinical applications in the realm of bone defect reconstruction.

## Materials and methods

### Cell culture

Bone marrow mesenchymal stem cells (BMSCs) were isolated from the femur and tibia of rats weighing 60–80 g, following previously established protocols [29]. Briefly, 10^5^ BMSCs were seeded in a 6 cm dish and cultured in exosome-free 10% FBS (Inner Mongolia Opcel Biotechnology Co. LTD, BS-1101), DMEM/F12 medium (Basal Media), 1% penicillin−streptomycin solution (P/S, BioChannel Biological Technology Co, Ltd.). The CELLSAVING was purchased from NCM Biotech (C40100, Suzhou, China).Sub-culturing of BMSCs was performed at 90% confluency, and experiments were conducted using passage 2–3 BMSCs.

For osteogenic differentiation, cells were cultured with osteogenic media (MUXMT-90,021, Cyagen, Guangzhou, China), and the media was replaced every 2 days.

### Exosome isolation and identification

BMSC cells were divided into two identical 6 cm dishes (Jet Biofil), for each dish, 10^5^ BMSCs were seeded. One dish was cultured in exosome-free 10% FBS (VivaCell Biosciences, C3801-0050) DMEM/F12 medium for 48 h, and the supernatant was collected (Exo-MSC) and froze in -80℃. Subsequently, the cells were washed three times with PBS and fresh exosome-free medium was added. Then, Exo-MSC supernatant from both 48 and 96 h were mixed and prepared for the isolation of Exosomes.

In another dish, cells were cultured in 10µM Yoda1 supplemented exosome-free 10% FBS (Procell Life Science &Technology Co., Ltd) DMEM/F12 medium for 48 h, and the supernatant was collected (Exo-Yoda1) and froze in -80℃. Subsequently, the cells were washed three times with PBS and fresh 10µM Yoda1 supplemented exosome-free medium was added. After an additional 48 h, the supernatant was collected. Then, Exo-Yoda1 supernatant from both 48 and 96 h were mixed and prepared for the isolation of Exosomes.

Exosomes, including Exo-MSCs derived from BMSCs and Exo-Yoda1 derived from Yoda1-treated BMSCs, were isolated using the ExoQuick exosome precipitation reagent, following previously described methods [30]. The isolated exosomes were diluted and quantified using the BCA Protein Assay Kit (Thermofisher). The concentration of exosomes we used in this work was calculated by protein concentration we measured by BCA Protein assay.

The identification of exosomes was performed using various techniques, including Transmission Electron Microscopy (TEM), nanoparticle tracking analysis (NTA), and Western blotting [31]. For the Western blot analysis, CD63 (Proteintech, 25682-1-AP), TSG101 (Proteintech,28283-1-AP), Calnexin (Proteintech, 10427-2-AP), and beta-Actin β-Actin (CST, 4967), Goat Anti-Rabbit (115-035-146, Jackson ImmunoResearch, USA) and Goat Anti-Mouse IgG (111-035-144, Jackson ImmunoResearch, USA) were used as specific markers.

For exosome endocytosis, Exo-MSC and Exo-Yoda1 were labeled using the DiI kit (Abcam, ab145311) following manufacturer’s instructions. The final concentration of each group was 100 µg/ml.The labeled Exo-MSCDil and Exo-Yoda1Dil were co-cultured with BMSCs for 4 h. After washing three times with PBS, the BMSCs were fixed using 4% PFA and stained with DAPI. Images were captured using the Leica DM3000. The Glass Bottom Cell Culture Dish for confocal microscope photography was purchased from SORFA Life Science (China).

### ARS and ALP staining

ALP staining (Yeasen, 21101ES60) was performed 5 days after differentiation with/without exosomes treating in cells. Alizarin red staining was performed as manufacture’s introduction (Yeasen,60504ES25) 14 days after differentiation in cells.

### Transwell assay

BMSCs were cultured in the upper chamber using exosome-free 10% FBS DMEM/F12 medium. For control group, 500 µl of medium was added to the lower chamber (NEST Biotechnology, 723,001). For Exo-MSC and Exo-Yoda1 groups, 500 µl of medium with a concentration of 100ug/ml exosomes was added to the lower chamber. For Yoda1-treated group, 500 µl of medium with a concentration of 10µM Yoda1 was added to the lower chamber. The BMSCs were cultured for 12 h, fixed, and stained with crystal violet dye. Images were taken using the Leica DM3000.

### Osteogenic differentiation and analysis

To induce osteogenic differentiation, cells were cultured with osteogenic media (MUXMT-90,021, Cyagen, Guangzhou, China). For control group, 10^6^ BMSCs were culture in osteogenic media. For Exo-MSC and Exo-Yoda1 groups, cells were culture in osteogenic media with a concentration of 100ug/ml exosomes. For Yoda1-treated group, cells were culture in osteogenic media with a concentration of 10µM Yoda1.

RNA was isolated from cell samples 5 days after differentiation using TRIZOL (Invitrogen). qPCR was performed using he SPARKscript II RT Plus kit (With gDNA Eraser) and SYBR Green qPCR Mix (with ROX) were purchased from the Shandong Sparkjade Biotechnology Co., Ltd. qPCR was performed on QuantStudio (Invitrogen). Primers for qpcr were listed in Table S1 (Supplementary S1). Protein aliquots, resolved in NP40 lysis buffer with Protease Inhibitor Cocktail (NCM Biotech, P001), were collected 7 days after differentiation. Western blotting was utilized to assess the expression of specific genes [29], the antibody that used in this work including the following: Collagen I (Abcam, ab260043), OPN (Abcam, ab214050)), ErK (CST, 4695), p-ErK (CST, 4370), α-Tubulin (CST, 3873), β-Actin (CST, 4967), Goat Anti-Rabbit (115-035-146, Jackson ImmunoResearch, USA) and Goat Anti-Mouse IgG (111-035-144, Jackson ImmunoResearch, USA) were applied. West Femto Maximum Sensitivity Substrate (34,094, Thermofisher, USA) was used to visualize the results. Biorad ChemiDoc system was used for imaging. Image J was used to export grey density analysis.

### Immunofluorescence staining

For cell Immunofluorescence staining in Fig. 2, BMSCs were cultured in osteogenic media for 7 days and fixed with 4% PFA. Immunofluorescence staining procedures were the same as we did previously [32]. The images were captured using Confocol.

Tissue samples were collected and fixed with 4% PFA for over 24 h. Using Freezing Microtome (Leica), slice of 5 μm were obtained. The staining of tissue samples were the same as cell samples. The following antibody were used: Collagen I (Abcam, ab260043), OCN (Proteintech, 23418-1-AP), CD31 (Proteintech,11265-1-AP), phalloidin (Beijing Solarbio Science &Technology Co., Ltd.), and HRP-conjugated secondary antibodies (Jackson ImmunoResearch). Additionally, 1% DAPI (Beijing Solarbio Science &Technology Co., Ltd.) was used to stain the cell nuclei. Finally, all the images were taken under a microscope (Leica DM3000).

### Transcriptome analysis

Exo-Yoda1 (100ug/ml), Yoda1(10µM Yoda1) or PBS were added into F12 10% FBS medium and cultured with 10^6^ cells for 24 h. RNA was isolated as described above. NanoDrop ND-1000 (NanoDrop, Wilmington, DE, USA) and Bioanalyzer 2100 (Agilent, CA, USA) was used for quality control. After fragment processing, the 2 × 150 bp paired-end sequencing (PE150) on an illumina Novaseq 6000 (LC-Bio Technology CO., Ltd., Hangzhou, China) was performed following the vendor’s recommended protocol. The differentially expressed mRNAs were selected with fold change > 2 or fold change < 0.5 and with parametric F-test comparing nested linear models (*p* value < 0.05). KEGG and GO analysis were conducted.

### Preparation of exosomes loaded GELMA / SAMA/β-TCP hydrogels

GELMA/SAMA/β-TCP hydrogels were prepared as described before [33]. 4wt% GELMA/1wt% SAMA/0.5wt% β-TCP hydrogel solution was prepared for bluelight curable printing. This liquid hydrogel was supplemented with 0.5% wt LAP, resulting in a final LAP concentration of 0.1% wt. Subsequently, exosomes were introduced into the solution at a final concentration of 1000 µg/ml. Thorough mixing ensued, followed by the injection to polytetrafluoroethylene mold with dimensions measuring 8 mm in diameter and 1 cm in height. Following a 1-minute exposure to irradiation (405 nm blue light), the hydrogels underwent curing and were rendered ready for use.

### Characteristics of GELMA / SAMA/β-TCP hydrogels

For scanning electron microscopy (SEM) analysis, the hydrogel samples, having been subjected to freeze-drying and molding, were subjected to brittle fracturing within a liquid nitrogen environment. The cross-sectional morphology of the material was then documented, employing specimens measuring 0.5 cm × 0.5 cm × 2 mm to 4 mm in size. Subsequently, a 30-second spray deposition period was applied, followed by microstructural examination of the scaffolds via a field emission scanning electron microscope. Specific imaging parameters included a projection height of 4 mm and a shooting voltage of 15 kV.

Regarding mechanical property evaluation, cylindrical hydrogel samples with dimensions of 10 mm in diameter and 5 mm in height were prepared. The assessment of hydrogel compressive strength was conducted employing a comprehensive mechanical testing apparatus, with a loading speed set at 1 mm/min. Each concentration of hydrogel underwent three separate tests, and subsequently, the maximum compressive strain and compressive strength of the hydrogel were determined based on strain and stress measurements at the point of hydrogel rupture. The calculation of compressive strength was executed as follows:

$${\sigma }= \frac{P}{\pi {r}^{2}}$$

For Rheological Property Analysis, we can record the storage modulus (G ‘) and loss modulus (G’ ‘) of hydrogel in diverse shear stress, time, frequency by rheometer. The status of hydrogels can be induced from these indexes. When G’< G”, the system approximates a viscous liquid, however, when G’> G”, the system approximates a solid-like colloid.

### Exosome sustained releasing assay

For exosomes sustained releasing assay in rats, DIR (Yeasen, 40757ES25) was used to labeled Exosomes. The labeled Exo-MSC DIR and Exo-Yoda1 DIR were mixed with hydrogels separately at a final concentration of 1000ug/ml. SD rats were anesthetized and incision were made on the skull top. 100 µl hydrogel solution (contained 100ug exosomes) was drip and cross-linked under 405 nm light. The skin was sutured after the hydrogel was solidified. Imaging analysis was performed using a Living Image Universal 0,1,3,7,14,21 days after operation.

### BMSCs osteogenic differentiation in vitro

For in vitro BMSCs culturing in gels, 100 µl hydrogels (contained 100ug exosome/PBS and 10^6^ cells) were prepared in molds, expose them to 405 nm blue light for 120 s, and then demold. After culturing with osteogenic media for 21 days and medium was changed every two days. Samples were collected for histological analysis.

Samples were fixed with 4% PFA for 24 h and and embedd in a paraffin block. The embedded specimens were cut into 5 μm-thick histological sections across the center of the defect area and stained with H&E. ARS was conducted as described above. Immunohistochemical staining was conducted as we conducted previously [33] .

### Construction and evaluation of subcutaneous ectopic osteogenesis model in nude mice

Subcutaneous ectopic osteogenesis models were constructed as described before [29].For each group, we prepared 6 mice. After the mice were anesthetized, three 0.5 cm incisions were made, and hemostatic forceps were used to create a subcutaneous cavity on the back of nude mice.

Hydrogels (100 µl contained 100ug exosome/PBS and 10^6^ cells) were prepared in molds (a syringe), expose them to 405 nm blue light for 120 s, and then demold by cutting 100 µl volume hydrogels using sterilized blade according to the scale of syringe. Gels were implanted subcutaneously. The incisions were sutured with absorbable 4 − 0 thread, and after disinfection with iodine, the nude mice were returned to the incubator and kept warm (37 ℃) with red light irradiation. After 30 days post-surgery, samples were collected for testing. Samples were fixed with 4% PFA for 24 h and embedd in a paraffin block. The embedded specimens were cut into 5 μm-thick histological sections Subsequently, H&E, Masson, ARS, and immunofluorescence staining were conducted. The 3D scans were conducted using CTvox.

### Construction and evaluation of skull defect model in SD rats

SD rats were anesthetized using pentobarbital and then randomly divided into three groups: Control, Exo-MSC, andExo-Yoda1 (*n* = 6). After the SD rats were anesthetized, a defect area with an approximate diameter of 4 mm was drilled. Hydrogels (100 µl contained 100ug exosome/PBS and 10^6^ cells) were applied to the defect areas and exposed to 405 nm blue light for 120 s to induce coagulation. The periosteum surrounding the defect areas was carefully covered, followed by suturing of the deep fascia and skin. After 4/8 weeks post-operation, the SD rats were anesthetized, and calvarial specimens were collected and fixed with 4% paraformaldehyde for further study. Micro-CT (SkyScan, SkyScan1176, Belgium) was initially used to assess the regeneration conditions in the defect area. H&E, Masson and and immunofluorescence staining techniques were also employed [29].

### Statistical analysis

GraphPad statistical software was used to analyze all the data. One way ANOVA was used to show the statistically significant difference of each set of data. The difference was considered statistically significant at *p* < 0.05.

### Electronic supplementary material

Below is the link to the electronic supplementary material.

Supplementary Material 1

## Data Availability

The data that support the findings of this study are available upon reasonable request from the corresponding authors.
