# Supplementary material for: Yoda1 pretreated BMSC derived exosomes accelerate osteogenesis by activating phospho-ErK signaling via Yoda1-mediated signal transmission
Source: J Nanobiotechnology. 2024 Jul 10;22:407. doi: 10.1186/s12951-024-02669-0 (PMC11234696; doi:10.1186/s12951-024-02669-0)
Supplement: Supplementary file 1 — Supplementary Material 1 [file 12951_2024_2669_MOESM1_ESM.docx]

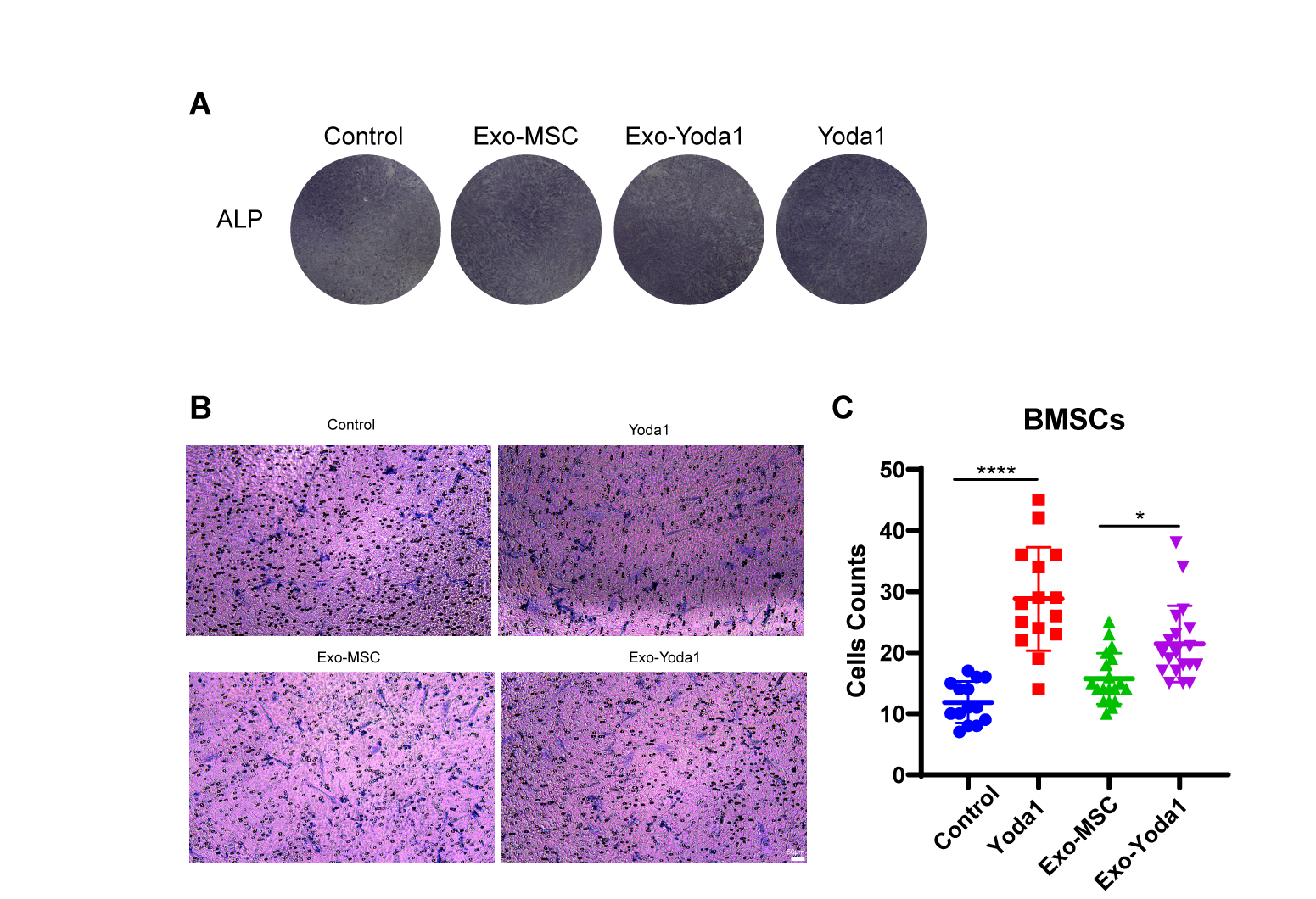


Figure S1 ALP staining and Transwell Assay

(A) Alizarin red (lower) staining of control and treated groups;(B) by Transwell Assay and crystal violet dying, migration was observed in Control, Exo-MSC, Exo-Yoda1, and Yoda1 groups, the scale was 50um; (C) the statistical analysis of migration, * p<0.5, **p<0.01, *** p<0.001, **** p<0.0001


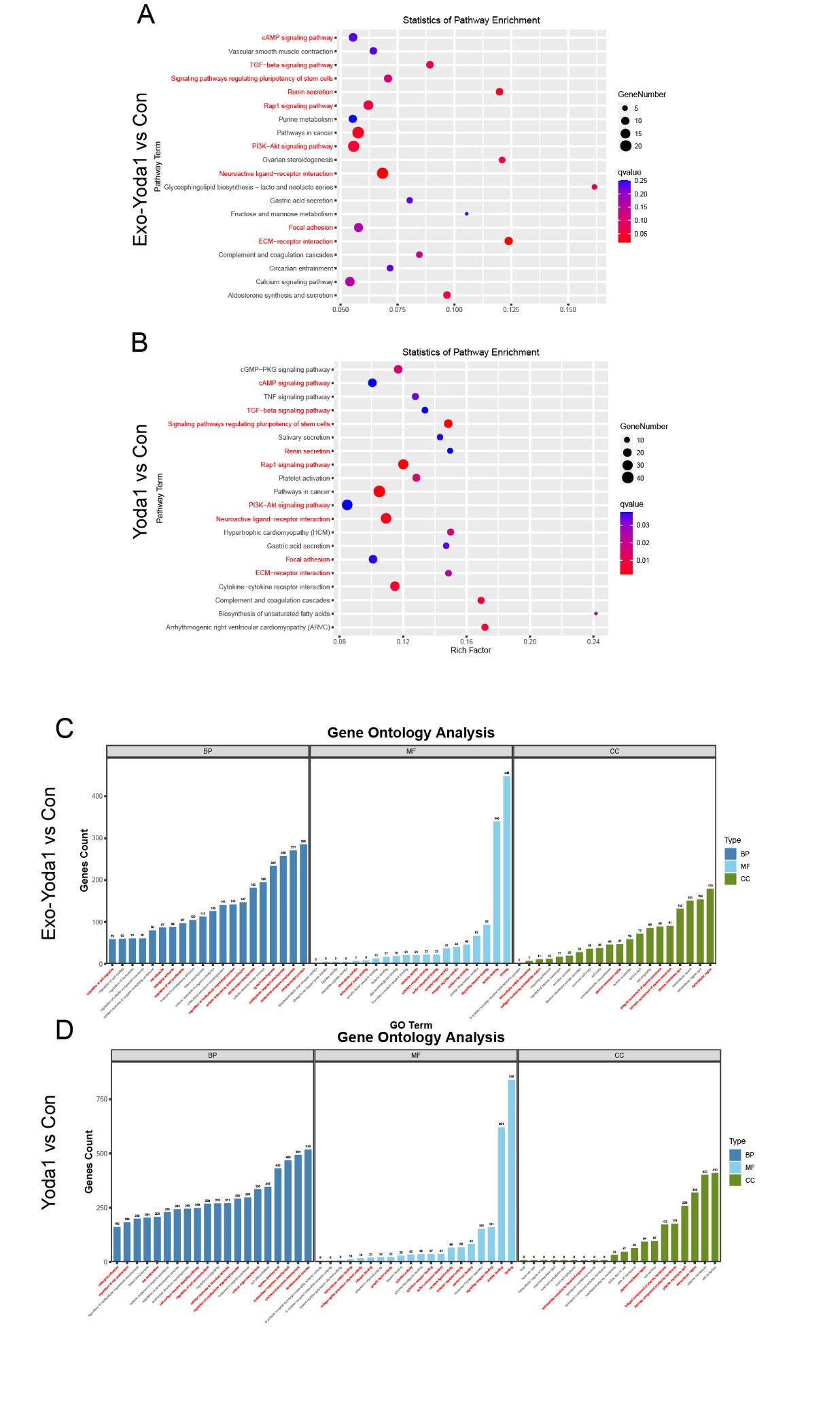


Figure S2 KEGG and GO analysis of Exo-Yoda1 and Yoda1 treated BMSCs

(A) and (B) KEGG analysis of Exo-Yoda1 and Yoda1; (C) andd (D) GO analysis of Exo-Yoda1 and Yoda1


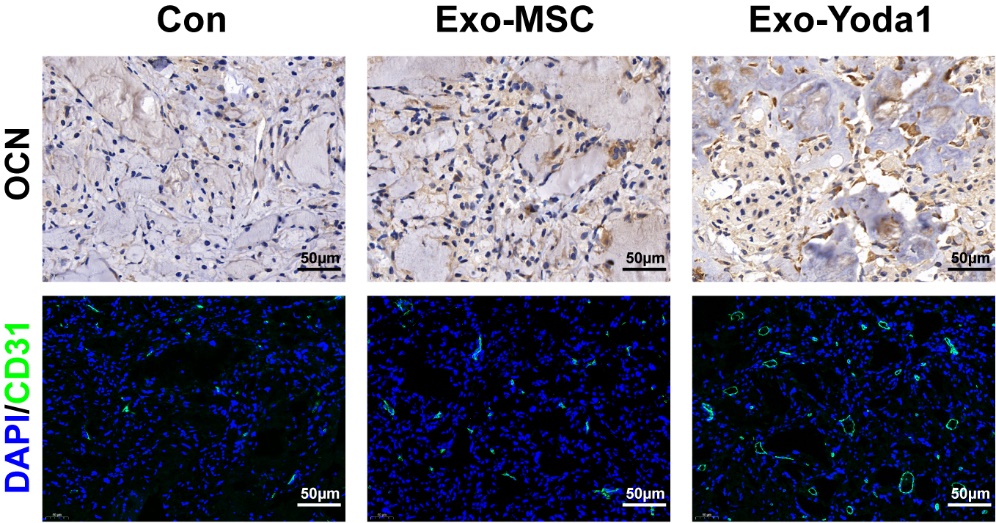


Figure S3 OCN and CD31(marker of vessels) staining in subcutaneous ectopic osteogenesis nude mice models


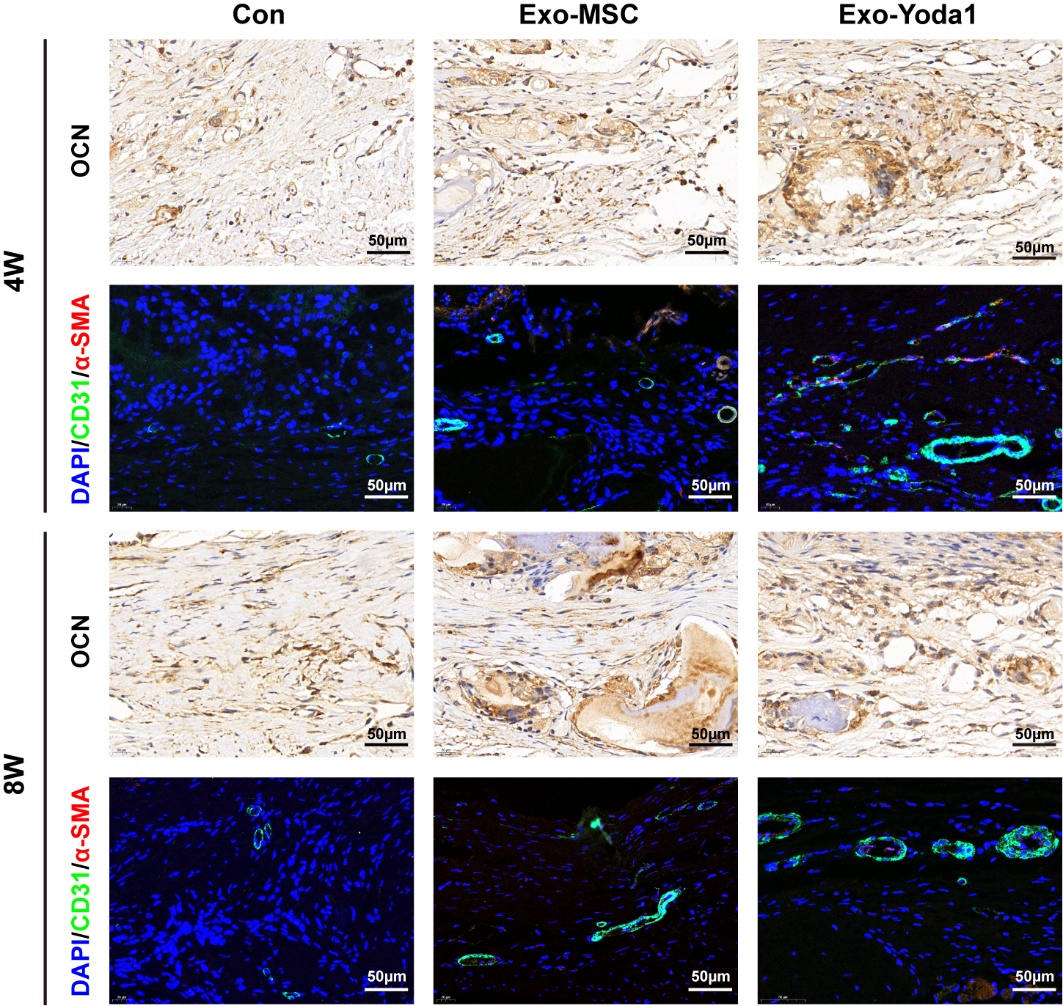


Figure S4 OCN and CD31(marker of vessels) staining in bone defect of rat skull models

| Primers | Sequence (5’-3’) |
| --- | --- |
| OPN-RAT-F | AGCTTGGCTTACGGACTGAG |
| OPN- RAT-R | AGGTCCTCATCTGTGGCATC |
| COL1A1-RAT-F | GTCATCGTGGCTTCTCTGGT |
| COL1A1- RAT-R | ACCGTTGAGTCCATCTTTGC |
| OCN-RAT-F | AATAGACTCCGGCGCTACCT |
| OCN- RAT-R | GAGCTCACACACCTCCCTGT |
| ALP-RAT-F | GCACAACATCAAGGACATCG |
| ALP- RAT-R | TCAGTTCTGTTCTTGGGGTACAT |
| BMP2-RAT-F | TGAACACAGCTGGTCTCAGG |
| BMP2- RAT-R | GCTGTTTGTGTTTGGCTTGA |
| Gapdh-Rat-F | TGGAGTCTACTGGCGTCTT |
| Gapdh-Rat-R | TGTCATATTTCTCGTGGTTCA |

Table S1 primers used in this study

Table S2 GO analysis of MAPK-ERK1/2 pathway

| Groups | Term | P value and q value | Genes |
| --- | --- | --- | --- |
| Exo-Yoda1 VS Con | GO:0070372  **regulation of ERK1 and ERK2 cascade** | p= 0.0000013, q= 0.000037066969282284 | Ackr3\|Angpt1\|Bmp4\|Ccl2\|Ccl7\|Ccr1\|Ceacam1\|Ceacam4\|Clcf1\|Cx3cl1\|Ednra\|Errfi1\|Fgf2\|Fgfr3\|Gcnt2\|Gcnt6\|Gper1\|Gpr183\|Kdr\|LOC100909750\|Nox4\|Pdgfd\|Pkhd1\|Spry2\|Xcl1 |
| Exo-Yoda1 VS Con | GO:0000165 MAPK cascade | p= 0.000011, q= 0.0001675958094383 | Ackr3\|Adra1b\|Angpt1\|Bmp4\|Bmp6\|Camk2a\|Ccl2\|Ccl7\|Ccr1\|Ceacam1\|Ceacam4\|Clcf1\|Cx3cl1\|Dact1\|Diras2\|Dusp2\|Dusp5\|Ednra\|Errfi1\|Fgd2\|Fgf2\|Fgfr3\|Gcnt2\|Gcnt6\|Gdf6\|Gper1\|Gpr183\|Grem1\|Grik2\|Id1\|Igfbp3\|Ighv\|Il1rn\|Inhbb\|Kdr\|LOC100909750\|LOC102549344\|Lpar2\|Lpar3\|Map3k7\|Mdfi\|Nox4\|Pdgfd\|Pkhd1\|Sfrp1\|Sh2d3c\|Spry2\|Xcl1\|Zfp622 |
| Yoda1 VS Con | GO:0070372  **regulation of ERK1 and ERK2 cascade** | p= 0.000000022, q= 7.89162481170248E-07 | Ackr3\|Angpt1\|Atf3\|Bmp4\|Btn2a2\|C5ar1\|C5ar2\|Ccl2\|Ccl7\|Ccr1\|Ceacam4\|Clcf1\|Cx3cl1\|Dusp1\|Ednra\|Epha7\|Errfi1\|Fgf10\|Fgf18\|Fgf2\|Fgfr3\|Gcnt2\|Gcnt6\|Gper1\|Gpr183\|Klf4\|LOC103689974\|Lgals9\|Mif\|Ndrg2\|Nox4\|P2ry1\|Pdgfd\|Rasgrp1\|Scimp\|Spry1\|Spry2\|Tgfbr3\|Trem2\|Wnk2\|Xcl1 |
| Yoda1 VS Con | GO:0000165 MAPK cascade | P= 0.0000000087, q= 3.61201261899261E-07 | Ackr3\|Actn2\|Adra1b\|Angpt1\|Atf3\|Bdnf\|Bmp3\|Bmp4\|Bmp6\|Btn2a2\|C5ar1\|C5ar2\|Cav2\|Ccl2\|Ccl7\|Ccr1\|Cd24\|Ceacam4\|Clcf1\|Cx3cl1\|Dact1\|Dusp1\|Dusp2\|Dusp5\|Dusp9\|Ednra\|Epha4\|Epha7\|Errfi1\|Fgf10\|Fgf14\|Fgf18\|Fgf2\|Fgfr3\|Flt1\|Fzd5\|Gcnt2\|Gcnt6\|Gdf6\|Gdnf\|Gper1\|Gpr183\|Grem1\|Id1\|Igfbp3\|Ighv\|Il11\|Il17rd\|Il1rn\|Inhba\|Inhbb\|Itga1\|Kit\|Klf4\|Kng1l1\|LOC100910021\|LOC100912585\|LOC102549344\|LOC103689974\|Lemd2\|Lgals9\|Lpar2\|Lpar3\|Map3k7\|Map3k7cl\|Mapk11\|Mdfi\|Mif\|Ndrg2\|Nox4\|Nrg1\|Nrk\|P2ry1\|Pdgfd\|Peli2\|Pik3r5\|Plce1\|Rasgrp1\|Rgs3\|Scimp\|Sfrp4\|Sh2d3c\|Spry1\|Spry2\|Tgfbr3\|Tnfrsf25\|Trem2\|Wnk2\|Wnt5a\|Xcl1 |
